# The Effect of Analogues of 1α,25-Dihydroxyvitamin D_2_ on the Regrowth and Gene Expression of Human Colon Cancer Cells Refractory to 5-Fluorouracil

**DOI:** 10.3390/ijms17060903

**Published:** 2016-06-14

**Authors:** Jacek Neska, Paweł Swoboda, Małgorzata Przybyszewska, Agnieszka Kotlarz, Narasimha Rao Bolla, Joanna Miłoszewska, Monika Anna Grygorowicz, Andrzej Kutner, Sergiusz Markowicz

**Affiliations:** 1 Department of Immunology, Maria Skłodowska-Curie Memorial Cancer Centre and Institute of Oncology, 5 WK Roentgen, 02-781 Warsaw, Poland; jacek.neska91@gmail.com (J.N.); pswoboda@o2.pl (P.S.); magip@coi.waw.pl (M.P.); akotlarz@coi.waw.pl (A.K.); joannam@coi.waw.pl (J.M.); m.a.grygorowicz@gmail.com (M.A.G.); 2 Chemistry Department, Pharmaceutical Research Institute, 8 Rydygiera, 01-793 Warsaw, Poland; n.bolla@ifarm.eu; 3 Pharmacology Department, Pharmaceutical Research Institute, 8 Rydygiera, 01-793 Warsaw, Poland; a.kutner@ifarm.eu

**Keywords:** 1α,25-dihydroxyvitamin D_2_, 1α,25-dihydroxyvitamin D_3_, vitamin D analogues, vitamin D and chemotherapy, cancer and vitamin D, cancer stem cell, colorectal cancer, HT-29 cell line

## Abstract

This study aimed to evaluate the capacity of hypocalcemic analogues of 1α,25-dihydroxyvitamin D_2_ (1,25D2) and 1α,25-dihydroxyvitamin D_3_ (1,25D3) to inhibit regrowth and regulate the stemness-related gene expression in colon cancer cells undergoing renewal after exposure to 5-fluorouracil (5-FU). All of the tested analogues of 1,25D2 equally potently decreased the clonogenicity and the proliferative activity of HT-29 cells which survived the exposure to 5-FU, but differently regulated gene expression of these cells during their renewal. 1,25D2 and analogues (PRI-1907 and PRI-1917), as well as 1,25D3 and analogue PRI-2191, decreased the relative expression level of several stemness-related genes, such as *NANOG*, *OCT3/4*, *PROM1*, *SOX2*, *ALDHA1*, *CXCR4*, in HT-29/5-FU cells during their renewal, in comparison to untreated HT-29/5-FU cells. The other 1,25D2 analogues (PRI-1906 and PRI-1916) were not capable of downregulating the expression of these stemness-related genes as the analogues PRI-1907 and PRI-1917 did. All of the tested vitamin D analogues upregulated *CDH1*, the gene encoding E-cadherin associated with epithelial phenotype. Out of the series of analogues studied, side-chain branched analogues of 1,25D2 (PRI-1907, PRI-1917) and the analogue of 1,25D3 (PRI-2191) might be used to target cancer cells with stem-like phenotypes that survive conventional chemotherapy.

## 1. Introduction

Colorectal cancer is the fourth cause of malignancy-related deaths worldwide [1]. Conventional chemotherapy regimens, such as 5-fluorouracil (5-FU)/leucovorin/oxaliplatin (FOLFOX), 5‑FU/leucovorin/irinotecan (FOLFIRI) or capecitabine/oxaliplatin (CapeOx), often fail to eradicate advanced colorectal cancer. It is assumed that to prevent cancer recurrence after conventional chemotherapy, new therapies should be developed to target cancer stem cells (CSC)/tumor initiating cells (TIC) that are more resistant to conventional cytoreductive drugs than differentiated cancer cells and are capable of initiating tumor regrowth [2,3,4]. So far, new therapeutic approaches have been focused on inducing differentiation of CSC [5,6] and sensitizing CSC for chemotherapy [7].

Active forms of vitamin D and its analogues have inhibitory effects on Wnt/β-catenin, Hedgehog, Notch and TGF-β signalling pathways, which are involved in self-renewal and differentiation of normal stem cells but are also aberrantly activated in different solid tumors [8]. Therefore, vitamin D and its analogues could be considered as candidate agents to target CSC/TIC, which initiate cancer recurrence after chemotherapy. Multiple studies suggest that vitamin D plays a role in cancer prevention [9,10,11,12,13]. A low level of vitamin D in the blood serum is associated with a higher risk of developing advanced adenomas [14]. There is a correlation between the low level of vitamin D and the risk of developing colorectal cancer [15,16,17,18]. Studies on mice showed that the administration of vitamin D analogues alone [19], and also in the combination with the cytoreductive drug 5-FU [20,21], retarded tumor growth. Although the combined treatment with cytostatics and vitamin D was more effective than the treatment with a cytostatic alone, the simultaneous administration of cytoreductive drugs and vitamin D in the combined treatment schedules led to increased toxicity [22,23,24]. Furthermore, vitamin D analogues block the cell cycle at the G1/S transition that prevents cancer cells from entering S-phase [20,24]. Cancer cells that are prevented by vitamin D from entering the S-phase of the cell cycle may become more resistant to cytoreductive drugs than rapidly dividing cells [20,24]. Since the combined use of vitamin D analogues and cytoreductive drugs can be associated with increased toxicity, a sequential administration of vitamin D analogues following the administration of cytoreductive drugs could be considered as an alternative treatment strategy aimed to counteract tumor regrowth after conventional chemotherapy. An additional argument in favor of the sequential vitamin D administration is based on the observation that the expression of stemness-related genes was highly increased in colorectal cancer cells that resisted the treatment with 5-FU in comparison to the chemonaive cells. Selected vitamin D analogues decreased the expression of stemness-related genes in 5-FU-pretreated colorectal cancer cells [25], PRI-2191 and PRI-1906 used at a very low concentration induced the loss of a stem-like phenotype and decreased the clonogenicity and proliferative activity of HT-29 cells undergoing renewal after exposure to 5-FU [25].

**Figure 1:**
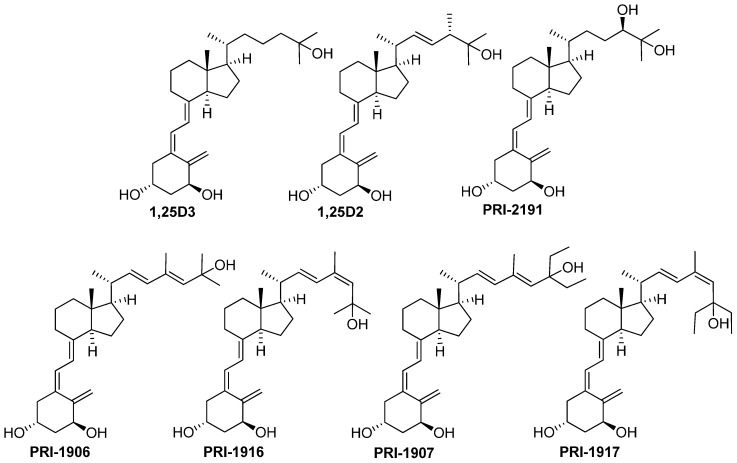
Structures of 1,25-dihydroxyvitamin D_3_ (1,25D3), 1,25-dihydroxyvitamin D_2_ (1,25D2), analogue of 1,25D3 (PRI-2191) and analogues of 1,25D2 (PRI-1906, PRI-1916, PRI-1907 and PRI-1917).

In our current efforts to optimize the structure of vitamin D analogues to increase their anti-cancer potential, we have compared the activity of our recently obtained side-chain modified geometric analogues of 1α,25-dihydroxyvitamin D_2_ (1,25D2), PRI-1916 and PRI-1917, with the previously obtained hypocalcemic analogues PRI-1906 and PRI-1907, as well with 1α,25-dihydroxyvitamin D_3_ (1,25D3) and its low-calcemic analogue PRI-2191 showing high anti-proliferative activity. The structures of vitamin D active metabolites and analogues are shown in Figure 1. Our present study was aimed at identifying vitamin D analogues optimally suited to counteract both the regrowth and stemness of colorectal cancer cells refractory to the treatment with 5-FU.

## 2. Results

### 2.1. The Effect of Vitamin D Analogues on the Clonogenicity of HT-29 Cells Preselected with 5-FU

**Figure 2:**
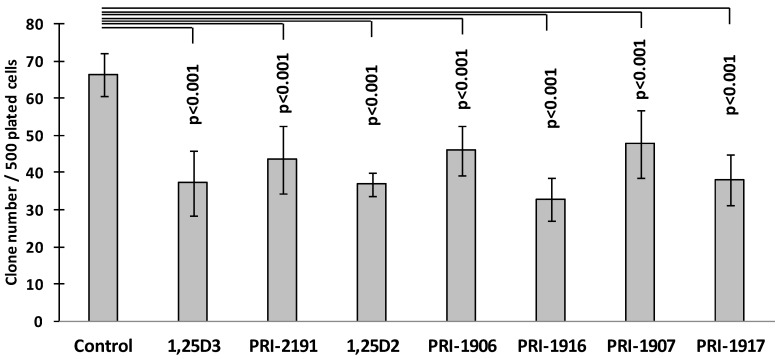
Vitamin D analogues decrease the clonogenicity of HT-29/5-FU cells undergoing renewal. The bar graph represents a mean colony numbers ± standard deviation in control cultures and in cultures with 1,25-dihydroxyvitamin D3 (1,25D3), analogue of 1,25D3 (PRI-2191) 1,25-dihydroxyvitamin D2 (1,25D2), and analogues of 1,25D2 (PRI-1906, PRI-1916, PRI-1907 and PRI-1917). HT-29/5-FU cells were collected on the third day after the exposure to 5-FU and were plated in the clonogenicity assay in *n* = 6 replicates. Statistically significant differences between mean colony-forming cell number in control cultures and cultures with vitamin D analogues were determined by Dunnett’s test. Data from one of two experiments with similar results are shown.

**Figure 3:**
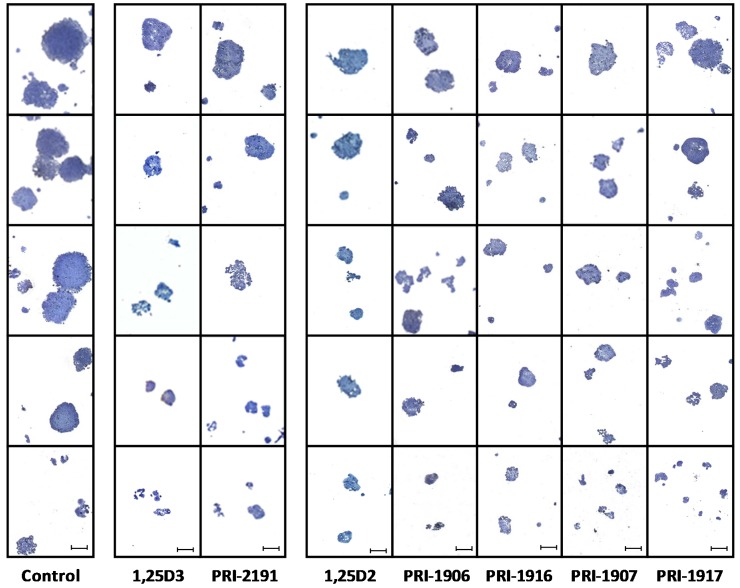
The size and appearance of colonies formed by HT-29/5-FU cells in the absence of the vitamin D (control), or in the presence of 1,25D3, PRI-2191, 1,25D2, PRI-1906, PRI-1916, PRI-1907 or PRI-1917. Photographs of the representative fixed colonies were taken on the 10th day of culture under the microscope at a 40-fold magnification. Five representative photographs shown for each culture type are placed in columns according to the decreasing size of colonies, starting from the top of each column. Scale bars representing 200 μm, shown only in the bottom row, apply to every photograph.

To select HT-29 cells refractory to 5-FU, HT-29 cells were cultured with 5-FU for 24 h, and then subsequently for three days in a culture medium without 5-FU. In the clonogenicity assay, HT-29 cells collected on the third day after 5-FU removal from the cell culture (HT-29/5-FU cells) were plated without or in the presence of vitamin D analogues. Both 1,25D3 and 1,25D2, as well as all the tested vitamin D analogues at a concentration of 100 nM, decreased the number of colonies formed by HT-29/5-FU cells (Figure 2) and reduced the yield of cells generated within colonies (Figure 3). HT-29/5-FU cells cultured in the presence of 1,25D3, 1,25D2 or their analogues formed mostly small colonies, whereas HT‑29/5-FU cells cultured in the absence of vitamin D formed mostly large colonies.

### 2.2. The Effect of Vitamin D Analogues on the Proliferative Activity and the Expression of Cancer Stem Cell (CSC) Surface Markers in HT-29 Cells Undergoing Renewal after Treatment with 5-FU

**Figure 4:**
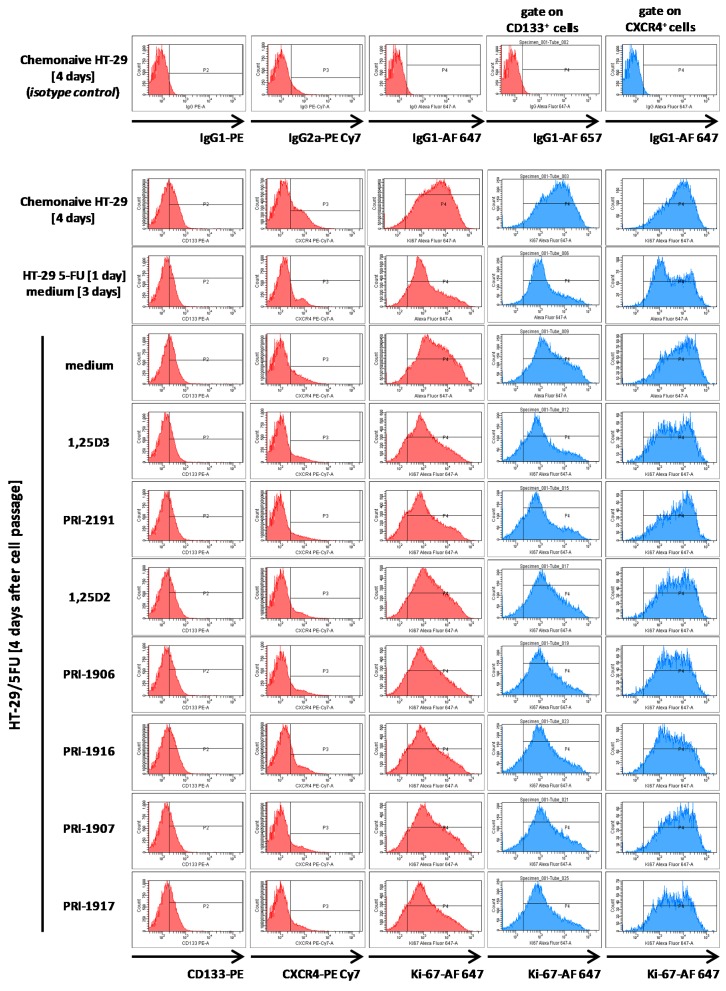
Flow cytometry analysis of CD133, CXCR4 and Ki-67 expression in chemonaive HT-29 cells, HT-29/5-FU cells on the third day after 5-FU removal from the culture, and in HT-29/5-FU cells passaged and cultured for four days with or without vitamin D analogues. CD133 and CXCR4 expression is shown for total chemonaive HT-29 cells and total HT-29/5-FU cell population. Ki-67 expression was analyzed in total cell populations and in gates set for CD133^+^ cells or CXCR4^+^ cells. Data from one out of three experiments with similar results are shown. Histograms showing analysis of samples labeled with fluorochrome-conjugated unspecific antibodies of respective isotypes, used as a negative control, are shown in the top row. Abbreviations: IgG—immunoglobulin G, AF 647—Alexa Fluor 647.

The exposure to 5-FU decreased the expression of CD133 and CXCR4 cell surface markers. Vitamin D analogues did not affect much the expression of CXCR4 and CD133 by HT-29/5-FU cells undergoing renewal during four-day culture after cell passage performed on the third day after 5-FU removal from the culture (Figure 4). The proliferative activity of HT-29/5-FU cells was evaluated on the basis of Ki-67 expression. Ki-67 protein is involved in ribosomal RNA transcription. Since Ki-67 is present at all the active cell cycle phases (G1, S, G2, and mitosis) but is strongly downregulated in resting cells (G0), Ki-67 is used as a cell proliferation marker [26,27]. Ki-67 expression decreased substantially in HT-29/5-FU cells on the third day after 5-FU treatment in comparison to chemonaive HT-29 cells. Ki-67 expression recovered during a four-day culture of HT-29/5-FU cells after cell passage. Both 1,25D3 and 1,25D2 inhibited the recovery of Ki-67 expression in HT-29/5-FU cells. Hypocalcemic PRI-2191 was as potent inhibitor of Ki-67 recovery as 1,25D3, and all of the tested analogues of 1,25D2 were at least as potent inhibitors of Ki-67 recovery as 1,25D2 itself.

The Ki-67 expression pattern in CD133^+^ subpopulation of chemonaive HT-29 cells was the same as in the total chemonaive HT-29 cell population, and the Ki-67 expression pattern in CD133^+^ subpopulation of HT-29/5-FU cells was the same as in total HT-29/5-FU cell population (Figure 4). Vitamin D analogues proportionally reduced Ki-67 expression level in CD133^+^ subpopulation of HT‑29/5-FU cells as in total HT-29/5-FU cell population. CXCR4^+^ HT-29/5-FU cell subpopulation contained a higher proportion of cells highly expressing Ki-67 than the total HT-29/5-FU cell population undergoing renewal. However, we found cells highly expressing Ki-67 not only in the CXCR4^+^ HT-29/5-FU cell subpopulation (Figure 4), but also in the CXCR4^−^ HT-29/5-FU cell subpopulation (data not shown). It suggests that neither CD133 nor CXCR4 expression allows for identification of cells univocally initiating renewal of HT-29 colon cancer cells after the treatment with 5-FU, both in the absence and in the presence of vitamin D analogues.

### 2.3. The Expression of Several Stemness-Related Genes Decreases in HT-29/5-FU Cells Undergoing Renewal in the Presence of Vitamin D Analogues

**Figure 5:**
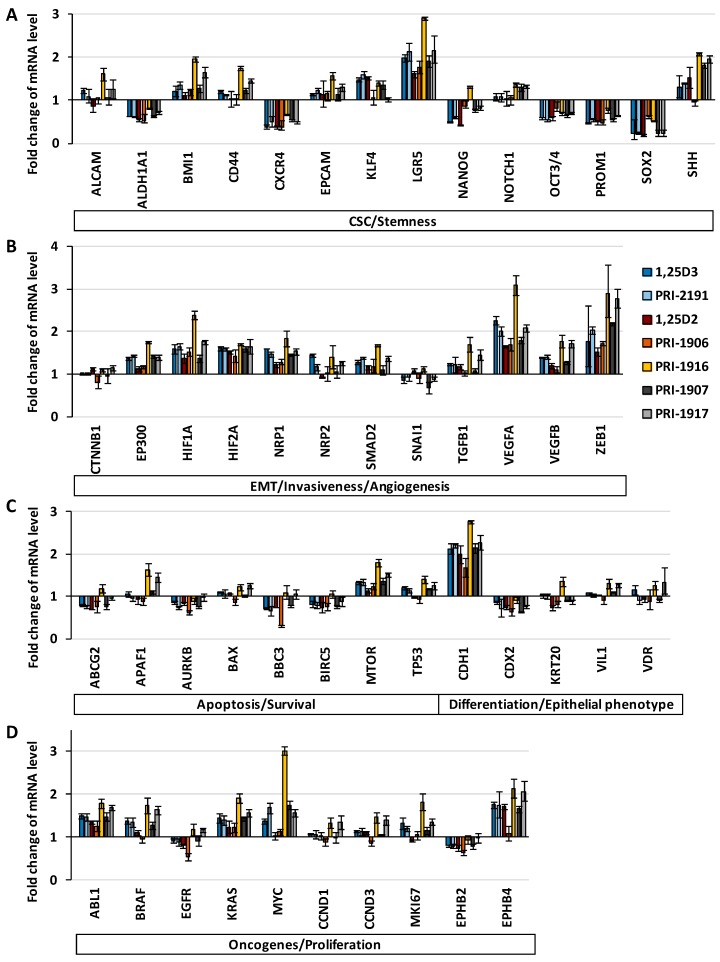
Comparison of the regulatory effects of 1,25D2, 1,25D3, and their analogues on gene expression in HT-29/5-FU cells undergoing renewal during four-day culture after cell passage. The graphs show fold change of the relative mRNA expression level of genes related to (**A**) cancer stem cells (CSC), stemness; (**B**) endothelial-mesenchymal transition (EMT), invasiveness, angiogenesis; (**C**) apoptosis, survival, differentiation and epithelial phenotype; (**D**) oncogenesis and proliferation in HT-29/5-FU exposed to vitamin D analogue (100 nM) for four days after cell passage, as compared to the level in HT-29/5-FU cells cultured in medium only for four days after cell passage. *GAPDH* was used as an endogenous control. Fold differences were calculated using the ΔΔ*C*_t_ method and are expressed as a range that is a result of incorporating the standard deviation of the ΔΔ*C*_t_ value into the fold-difference calculation.

The relative messenger RNA (mRNA) expression level of several groups of genes was analyzed in HT-29/5-FU cells undergoing renewal in the absence or in the presence of 1,25D3 or 1,25D2, or their analogues during four-day culture after cell passage. The mRNA expression analysis with the use of *GAPDH* as a reference gene showed that vitamin D analogues at a concentration of 100 nM modified the expression of genes during four-day culture of HT-29/5-FU cells after cell passage. However, certain groups of genes were differently regulated by vitamin D analogues (Figure 5).

The relative expression level of several stemness-related and cancer stem cell (CSC)-related genes, such as *NANOG*, *OCT3/4*, *PROM1*, *SOX2*, *ALDHA1*, *CXCR4*, was lower in HT-29/5-FU cells undergoing renewal in the presence of 1,25D3 or 1,25D2, or their analogues, than in HT-29/5-FU cells not exposed to vitamin D. However, vitamin D analogues augmented the expression of epithelial tissue stem cell-related genes *LGR5* and *SHH*. 1,25D3, 1,25D2 and their analogues increased the relative expression level of some genes associated with invasiveness and angiogenesis, but mostly did not substantially affect the relative expression level of genes associated with cell proliferation, cell differentiation, epithelial-mesenchymal transition (EMT), apoptosis and survival. PRI-1916 less potently downregulated the expression of stemness-related genes in comparison to the other vitamin D analogues (Figure 5), but more potently upregulated the expression of *LGR5*, oncogene *MYC*, and also particular genes associated with invasiveness and angiogenesis (*HIF1A*, *VEGFA*) and with cell survival (*MTOR*). All the tested vitamin D analogues upregulated *CDH1*, the gene encoding E-cadherin, a characteristic marker of epithelial phenotype.

**Figure 6:**
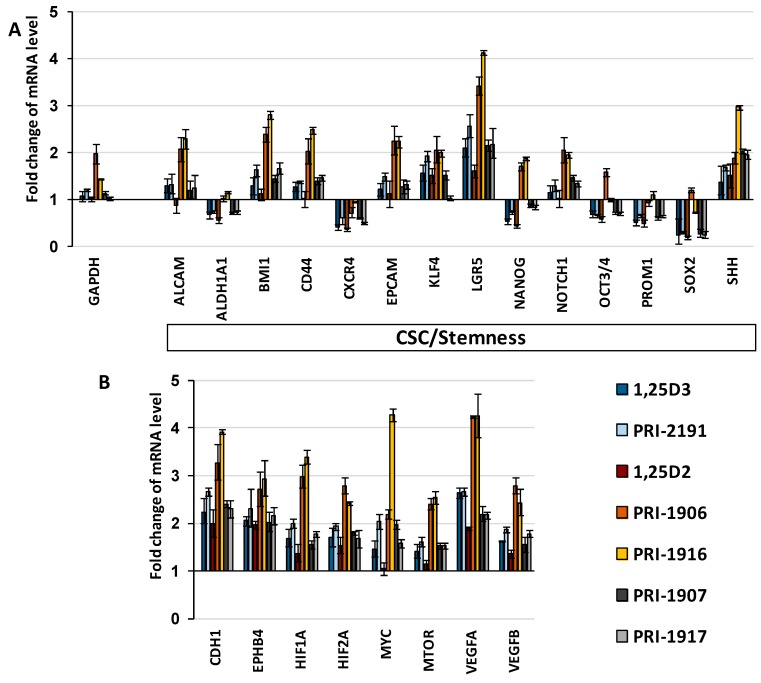
(**A**) gene expression analysis, performed with the use of *18S RNA* as an endogenous control, revealed that both PRI-1906 and PRI-1916 differ from 1,25D2, PRI-1907, PRI-1917, 1,25D3 and PRI-2191 in the capability to regulate the expression level of CSC/stemness-associated genes in HT-29/5-FU cells undergoing renewal during four-day culture after cells passage and (**B**) more potently increased the relative expression level of several other genes. Fold differences were calculated using the ΔΔ*C*_t_ method and are expressed as a range that is a result of incorporating the standard deviation of the ΔΔ*C*_t_ value into the fold-difference calculation.

We found that the relative *GAPDH* expression level, analyzed with the use of *18S RNA* as a reference gene, increased moderately in HT-29/5-FU cells on the third day after 5-FU removal from cell cultures in comparison to chemonaive HT-29 cells, but on the fourth day after cell passage, returned to the same level as observed in chemonaive HT-29 cells (data not shown). The relative *GAPDH* expression level was the same in HT-29/5-FU cells undergoing renewal in the absence of vitamin D as in HT-29/5-FU cells exposed to vitamin D analogues, except for PRI-1906 and PRI-1916 (Figure 6A). The relative expression level of *GAPDH* was two-fold higher in HT-29/5-FU cells undergoing renewal in the presence of PRI-1906 in comparison to HT-29/5-FU cells cultured without vitamin D, whereas we found only a minor effect of PRI-1916 on the *GAPDH* expression level.

The use of *GAPDH* as a reference gene in gene expression analysis could bias the evaluation of the PRI-1906 effect on the relative gene expression of the other genes due to the upregulation of *GAPDH* expression by PRI-1906. Therefore, we analyzed the relative mRNA expression also with the use of *18S RNA* as an endogenous control. Except for PRI-1906, the patterns of the relative fold change of mRNA levels in HT-29/5-FU cells cultured with vitamin D analogues, as compared to HT-29/5-FU cells cultured in the absence of the vitamin D, were basically analogous, if either *GAPDH* or *18S RNA* served as an endogenous control.

The use of *18S RNA* as a reference gene revealed that PRI-1906, similar to PRI-1916, did not match the capacity of 1,25D3, PRI-2191, 1,25D2, PRI-1907 and PRI-1917 to downregulate the relative expression level of stemness-associated genes *ALDH1A1*, *CXCR4*, *NANOG*, *OCT3/4*, *PROM1* and *SOX2* (Figure 6A). In addition, both PRI-1906 and PRI-1916 more potently upregulated the relative expression level of *ALCAM*, *BMI1*, *CD44*, *EPCAM*, *LGR5* and *NOTCH1* than the other tested vitamin D analogues, and upregulated the relative expression level of *NANOG*. If *18S RNA* served as an endogenous control in the gene expression analysis, PRI-1906 augmented the expression of such genes as *HIF1A*, *HIF2A*, *MTOR*, *VEGFA*, *VEGFB* and *CDH1* more efficiently than 1,25D2 and its analogues PRI-1907 and PRI-1917, but similarly to PRI-1916 (Figure 6B).

## 3. Discussion

Cancer stem cells are relatively more resistant to cytoreductive drugs than more differentiated cells; therefore, they are preferentially spared by conventional chemotherapy [28,29,30,31]. Furthermore, due to cancer cell plasticity, heterogeneous cancer cells acquire stem-like properties in response to cytoreductive agents [4,32,33,34]. Therefore, new agents should be found which could target cancer cells displaying stem-like features maintained or acquired after conventional chemotherapy. In this study, we showed that the anti-cancer activity of vitamin D analogues of 1,25D3 (PRI-2191), as well as of 1,25D2 (PRI-1907 and PRI-1917) encompassed both the anti-proliferative effect and the modification of stemness-associated gene expression, leading to a shift from the stem-like phenotype toward a more differentiated phenotype. PRI-2191, PRI-1907 and PRI-1917 downregulated the expression of *NANOG*, *OCT3/4* and *SOX2*, *i.e*., pluripotent stem cell-related genes involved in the stem cell renewal, which are aberrantly overexpressed in several cancers [35,36]. Stemness-associated genes *NANOG*, *OCT3/4* and *SOX2* are involved in self-renewal activity of colorectal cancer cells [37]. It can be speculated that the downregulation of *NANOG*, *OCT3/4* and *SOX2* expression by PRI-2191, PRI-1907 or PRI-1917 might decrease the capacity of residual CSC to initiate cancer renewal after conventional chemotherapy.

We found that PRI-2191, PRI-1907 and PRI-1917 decreased *ALDHA1* expression in HT-29/5-FU cells undergoing renewal. Aldehyde dehydrogenase 1 (ALDH1) is a marker for identification and tracking of colorectal CSC [38,39]. ALDH1 oxidizes intracellular aldehydes and thereby is involved in the resistance to alkylating agents [28]. High ALDH1 expression correlates with a poor clinical outcome [40,41]. ALDH1 expression induced after the chemotherapy may allow the acquisition of chemoresistance in residual cancers [42]. Our data suggest that PRI-2191, PRI-1907 and PRI-1917 might decrease chemoresistance of residual colon cancer cells after the chemotherapy due to the downregulation of *ALDHA1* expression.

In our study, PRI-2191, PRI-1907 and PRI-1917 decreased the expression of *CXCR4* and *PROM1* encoding CSC markers CXCR4 and CD133, respectively. The stromal cell-derived factor-1 (SDF-1/CXCR4) axis is involved in delivering signals related to chemotaxis, cell survival and/or proliferation, and thereby plays an important role in EMT and tumor invasiveness [43,44]. The co-expression of CXCR4 and CD133 in colon cancer may be associated with unfavorable prognosis [45,46]. Our data show that PRI-2191, PRI-1907 and PRI-1917 might be used to induce the loss of stem-like phenotype, as well as to decrease chemotactic and proliferative activity of colon cancer cells refractory to the cytoreductive treatment.

All of the tested vitamin D analogues upregulated *KLF4*. Krüppel-like factor 4 (KLF4) is a zinc finger transcription factor that functions as a tumor suppressor or as an oncogene. In colon cancer stem cell-enriched spheroid cells, KLF4 acts as an oncogene [47]. However, loss of KLF4 was associated with worse disease-free survival in colon cancer patients [48]. KLF4 directly represses *BMI1* transcription, and thereby regulates BMI1, which is required for colon cancer proliferation [49]. In our study, PRI-1906 and PRI-1916, but not other vitamin D analogues, substantially upregulated *BMI1*. In several carcinomas, including colon cancer, an overexpression of *BMI1* is related to cancer aggressiveness [50,51,52].

The tested vitamin D analogues augmented the expression of epithelial tissue stem cell-related genes *LGR5* and *SHH*. Reports on LGR5’s role in the survival and proliferation of colon cancer cells are confusing. The maintenance of CSC in spheroids required LGR5 activity [53]. On the other hand, LGR5 was reported as a negative regulator of tumorigenicity due to antagonizing Wnt signalling. Paradoxically, LGR5 ablation increased colon cancer cell invasiveness and xenograft tumorigenicity [54]. Hedgehog signalling is involved in differentiation and renewal of the colonic lining epithelium rather than in cancer formation. It was reported that the viability and proliferation of well-differentiated HT-29 adenocarcinoma cell line depends on active Hedgehog signalling [55].

Since the tested vitamin D analogues upregulate *CDH1*, they could be used to promote mesenchymal-to-epithelial transition and differentiation of residual colon cancer after conventional chemotherapy. It was reported that vitamin D3 promotes the differentiation of colon cancer cells by the induction of E-cadherin [5]. The tested vitamin D compounds, except for 1,25D2, upregulated *MYC* expression in spite of the concomitant upregulation of *CDH1*. Overexpression of c-MYC induces a neoplastic transformation in many types of cells [56]. However, c-MYC is a factor that has been shown to regulate stem cell exit from the epidermal stem cell compartment to become transit-amplifying cells that can undergo terminal differentiation [57].

Vitamin D analogues upregulated *EPHB4*. The receptor tyrosine kinase EPHB4 has oncogenic activities in various cancers. In colorectal cancer patients, low tumor EPHB4 levels are associated with poor prognosis [58]. In animal models, EPHB receptors regulate cell proliferation and migration in the intestine. EPHB4 has tumor suppressor activities in intestinal tumorigenesis [59,60].

We found that the effect of vitamin D analogues on the expression of stemness-associated genes may promote drift of colon cancer cells from the stem-like phenotype to the more differentiated phenotype. The effect of vitamin D analogues on the gene expression in colon cancer cells undergoing renewal after cytoreductive treatment was ambiguous in some aspects. Although vitamin D analogues enhanced the expression of some genes related to angiogenesis and proliferation, in fact, the anti-proliferative effects of vitamin D prevailed over pro-proliferative effects in colon cancer cell cultures.

The selected analogue of 1,25D3 (PRI-2191), which previously showed hypocalcemic activity [61], retained the potency of 1,25D3 to inhibit the proliferative activity of colon cancer cells. All of the tested analogues of 1,25D2, regardless of their side-chain length and geometry, were at least as potent inhibitors of the proliferative activity as the 1,25D2 itself. We found that PRI-1907 and PRI-1917 compared to 1,25D2, as well as PRI-2191 compared to 1,25D3, and similarly 1,25D2 compared to 1,25D3, did not differ in their capacity to regulate the relative gene expression level in colon cancer cells undergoing the renewal after the treatment with 5-FU. PRI-1906 and PRI-1916 differed from 1,25D2 and the other tested analogues in their effect on the relative expression level of stemness-related genes and particular genes associated with angiogenesis, invasiveness and proliferation in such cells. The effects of PRI-1906 and PRI-1916 on all of the tested genes were similar, except for *GAPDH*, *SHH*, *MYC* and *OCT3/4*. Our data suggest that the differences in the structure of vitamin D analogues are manifested in their different effects on the expression of genes that are involved in the maintenance of a stem-like phenotype in colon cancer cells refractory to cytoreductive treatment.

## 4. Materials and Methods

### 4.1. Drugs and Analogues

5-Fluorouracil (5-FU, Accord Healthcare Limited, North Harrow, UK) solution (50 μg/mL) was diluted in cell culture medium shortly before use. Diluted solution of 5-FU was added to cell culture at a final concentration of 6 μg/mL (46 μM). 1,25D3, 1,25D2, PRI-2191 [(24*R*)-1,24-dihydroxyvitamin D_3_, tacalcitol], PRI-1906, PRI-1907, PRI-1916, and PRI-1917 were synthesized at the Pharmaceutical Research Institute, Warsaw, Poland [62,63,64,65]. Samples of analogues were aliquoted to amber ampoules and dried down in a stream of argon. The samples were dissolved in absolute ethanol (99.8%) to obtain 20 μM stock solution. The solution was stored at −20 °C and diluted in a culture medium to a working concentration right before use.

### 4.2. Cell Line

The moderately differentiated human colon adenocarcinoma cell line HT-29 was purchased from Leibniz-Institut DSMZ-Deutsche Sammlung von Mikroorganismen und Zellkulturen GmbH (DSMZ, Braunschweig, Germany). Cells were cultured in McCoy’s 5a culture medium (Lonza, Basel, Switzerland) supplemented with glutamine, 10% inactivated FBS (Biowest, Nuaillé, France) and antibiotics. Cells were cultured in humidified atmosphere at 37 °C and 5% CO_2_.

### 4.3. Selection of Cancer Cells Refractory to Treatment with 5-FU

HT-29 cells were seeded in 75-cm^2^ flasks (Nunc, Rochester, NY, USA) at a cell density 85 × 10^3^ cells/mL of culture medium. After 4 h incubation, 5-FU was added at a working concentration of 6 μL/mL (46 μM). Cells were exposed to 5-FU for 24 h, and then the medium was replaced with a fresh medium without 5-FU. On the third day of culture after 5-FU removal, cells refractory to a single 5-FU treatment were collected using trypsin with EDTA (Trypsin-EDTA, Lonza, Basel, Switzerland). The collected cells were denominated as HT-29/5-FU cells.

### 4.4. Cultures of Cells Preselected with 5-FU

HT-29/5-FU cells collected on the third day after 5-FU removal from the culture were seeded in 75-cm^2^ flasks in the absence or in the presence of 1,25D3, PRI-2191, 1,25D2, PRI-1906, PRI-1916, PRI-1907 or PRI-1917, each added at a concentration of 100 nM. Fresh medium with or without vitamin D or analogues was replaced after 2 days. On the fourth day of the culture, cells were harvested using non-enzymatic cell dissociation solution Cellstripper^®^ (Corning, Manassas, VA, USA).

### 4.5. Clonogenicity Assay

HT-29/5-FU cells collected on the third day after 5-FU removal from the culture were plated on 35 mm diameter Petri dishes at a concentration of 5 × 10^2^ cells/dish, with or without 1,25D3, PRI-2191, 1,25D2, PRI-1906, PRI-1916, PRI-1907 or PRI-1917, added at a concentration 100 nM. On the 10th day of culture, cell colonies were fixed and stained with crystal violet in methanol. Colonies were counted under a microscope at a 100-fold magnification.

### 4.6. Antibodies and Flow Cytometry

PE-Cy7-anti-CXCR4 and Alexa-Fluor-647-anti-Ki67 was purchased from BD Biosciences (San Jose, CA, USA). PE-CD133/1 (AC133) was purchased from Miltenyi Biotec (Cologne, Germany). Following the labeling of cells with fluorochrome-conjugated monoclonal antibodies specific for extracellular markers, intracellular staining was performed using FoxP3 Staining Buffer Set (eBioscience, San Diego, CA, USA) according to the manufacturer’s protocol. Flow cytometry was performed using the FACSAriaIII (BD Biosciences). Data was analyzed using FACSDiva software (BD Biosciences).

### 4.7. Gene Expression Analysis

Total RNA was extracted using TRI Reagent Solution (Ambion, Carlsbad, CA, USA) according to the manufacturer’s protocol. Total RNA was transcribed to complementary DNA using high-capacity cDNA reverse transcription kit (Applied Biosystems, Foster City, CA, USA). Quality of the RNA was confirmed with a NanoDrop ND-1000 spectrophotometer (NanoDrop, Wilmington, DE, USA). The relative quantification of gene expression was analyzed in triplicates by TaqMan Array and a 7500 fast real-time PCR system (Applied Biosystems, Foster City, CA, USA). The *C*_t_ comparative method was used with *GAPDH* or *18S RNA* serving as an endogenous control. Data output was expressed as a fold-change of expression levels. Fold differences were calculated using the ΔΔ*C*_t_ method, expressed as a range that is a result of incorporating the standard deviation of the ΔΔ*C*_t_ value into the fold-difference calculation.

### 4.8. Statistics

Statistical significance was evaluated with one-way ANOVA with Bonferroni multicomparison post-test correction and with Dunnett’s test using SPSS 14.0 (SPSS Inc., Kraków, Poland). *p*-Values < 0.05 were considered significant.

## 5. Conclusions

Our data suggest that the analogues of 1,25D2, PRI-1907 and PRI-1917, and the analogue of 1,25D3 (PRI-2191), could be considered in further studies as candidate compounds to counteract cancer recurrence by decreasing the proliferative capacity of cells initiating tumor regrowth and by downregulation of the stemness-related genes, if applied sequentially after the treatment with conventional cytoreductive chemotherapy. PRI-1906 and PRI-1916 inhibit the regrowth of colon cancer cells refractory to the treatment with 5-FU, but do not share the capability of the other tested analogues (PRI-1907 and PRI-1917) to induce the downregulation of the stemness-related genes.

## Abbreviations

5-FU	5-Fluorouracyl
AF 647	Alexa Fluor 647
CapeOx	Capecitabine/oxaliplatin
CSC	Cancer stem cells
EMT	Epithelial-Mesenchymal Transition
FBS	Foetal bovine serum
FOLFIRI	Leucovorin, 5-Florouracil, Irinotecan
FOLFOX	Leucovorin, 5-fluorouracyl, Oxaliplatin
HT-29/5‑FU cells	HT-29 colon cancer cells preselected with 5-FU
IgG	Immunoglobulin G
TIC	Tumor initiating cells
